# Functional aggregation of cell-free proteins enables fungal ice nucleation

**DOI:** 10.1073/pnas.2303243120

**Published:** 2023-11-09

**Authors:** Ralph Schwidetzky, Ingrid de Almeida Ribeiro, Nadine Bothen, Anna T. Backes, Arthur L. DeVries, Mischa Bonn, Janine Fröhlich-Nowoisky, Valeria Molinero, Konrad Meister

**Affiliations:** ^a^Department of Molecular Spectroscopy, Max Planck Institute for Polymer Research, Mainz 55128, Germany; ^b^Department of Chemistry, The University of Utah, Salt Lake City, UT 84112; ^c^Multiphase Chemistry Department, Max Planck Institute for Chemistry, Mainz 55128, Germany; ^d^Department of Animal Biology, University of Illinois at Urbana-Champaign, Urbana, IL 61801; ^e^Department of Chemistry and Biochemistry, Boise State University, Boise, ID 83725

**Keywords:** heterogeneous ice nucleation, protein assembly, fungi, ice-nucleating proteins

## Abstract

Biological ice nucleation plays a key role in the survival of cold-adapted organisms. Several species of bacteria, fungi, and insects produce ice nucleators (INs) that enable ice formation at temperatures above −10 °C. Bacteria and fungi produce particularly potent INs that can promote water crystallization above −5 °C. Bacterial INs consist of extended protein units that aggregate to achieve superior functionality. Despite decades of research, the nature and identity of fungal INs remain elusive. Here, we combine ice nucleation measurements, physicochemical characterization, numerical modeling, and nucleation theory to shed light on the size and nature of the INs from the fungus *Fusarium acuminatum*. We find ice-binding and ice-shaping activity of *Fusarium* IN, suggesting a potential connection between ice growth promotion and inhibition. We demonstrate that fungal INs are composed of small 5.3 kDa protein subunits that assemble into ice-nucleating complexes that can contain more than 100 subunits. *Fusarium* INs retain high ice-nucleation activity even when only the ~12 kDa fraction of size-excluded proteins are initially present, suggesting robust pathways for their functional aggregation in cell-free aqueous environments. We conclude that the use of small proteins to build large assemblies is a common strategy among organisms to create potent biological INs.

## Significance

Organisms have evolved efficient molecular strategies to control the nucleation and growth of ice. Although these strategies have developed independently across biological kingdoms, they all seem to use proteins to construct extended functional domains. While for bacteria, the use of large proteinaceous units to build superior ice-making complexes is established, the identity of fungal INs remains unknown. Here, we demonstrate that small extracellular proteins, of which over a hundred are capable of assembling in cell-free environments, make up the fungal INs that enable ice formation at warm temperatures. Our findings highlight that nature uses a common strategy, *E pluribus unum* (out of many, one), to enable high subzero ice nucleation temperatures by assembly of ice-nucleating proteins into large functional aggregates.

The crystallization of water is the most prevalent liquid-to-solid phase transition on Earth. Ice formation is thermodynamically favored at temperatures below 0 °C, but the crystallization process is kinetically hindered by the cost of the ice nucleus interface. Consequently, pure water microdroplets can be supercooled to temperatures as low as −46 °C, below which homogenous ice nucleation seems to be unavoidable (1). In nature, the freezing of water is usually a heterogeneous process facilitated by ice nucleators (INs) of biological and abiotic origins. Naturally occurring abiotic INs typically elevate freezing temperatures to −15 to −30 °C, whereas biological INs are more active and proteinaceous INs can facilitate freezing at temperatures between −2 and −15 °C (2). The ecological advantages of ice nucleation and its potential impact on cloud glaciation and precipitation are not yet fully understood and constitute a significant gap in our understanding of the relationship between climate and life.

The best-characterized biological INs are plant-associated bacteria of the genera *Pseudomonas, Pantoea*, and *Xanthomonas,* which enable ice formation at temperatures close to 0 °C (3). The ability of the ice-nucleation-active bacteria to facilitate ice formation is attributed to ~120 kDa ice-nucleating proteins (INPro) that are anchored to their outer cell membrane and that form functional aggregates to achieve activity at high temperatures (4–6). The gene encoding the large bacterial INPro monomers has been identified and found to be conserved across diverse ice nucleation-active gram-negative bacteria (7, 8).

Fungi also produce very effective biological INs, enabling the crystallization of water at temperatures as warm as −2 °C (9, 10). The ice nucleation activity in fungi was first discovered in the genus *Fusarium* and later observed in multiple other genera (e.g., *Isaria, Mortierella, Sarocladium, Puccinia*) (11–13). The cosmopolitan genus *Fusarium* comprises saprophytes and pathogens of plants and animals, and it is the most studied ice nucleation active fungus (9, 10). Although widely distributed in soil and on plants, it has also been detected in atmospheric and cloud water samples, making it a highly relevant biological and atmospheric model system (11, 14). Both ice nucleation active bacteria and fungi can cause frost damage to plants, and their presence in precipitation samples has led to suggestions that these biological INs may influence regional and global weather patterns (15–18).

However, unlike the bacterial INPro (3, 4, 19), the chemical composition and structures of the macromolecules responsible for ice nucleation activity in fungi are unknown. Our current understanding suggests that *Fusarium* INs are cell-free secreted soluble macromolecules that are stable at pH values from 2 to 12 (9, 10, 20, 21). Kunert et al. determined the size of *Fusarium* INs to be <100 kDa based on molecular weight cutoff (MWCO) filtration experiments (10). Similarly, Yang et al. utilized MWCO filters and determined that the size of fungal INs is <30 kDa (21). However, the use of MWCO filters has limitations, as these filters only provide nominal classifications and not precise boundaries. Achieving 100% retention, even for very large molecules, is not possible and drawn conclusions can be ambiguous. The *Fusarium* INs were proposed to be at least partially proteinaceous, given their heat inactivation, peak UV absorbance at 280 nm, and sensitivity to certain proteinases (10, 21, 22). In addition, Vinatzer and coworkers recently employed comparative genomics and transcriptomics to identify over 200 candidates for ice nucleation genes that code for secreted proteins preferentially expressed at low temperatures (21). However, the assumption that ice nucleation sites of biological origin are denatured by heat while those associated with abiotic nucleators are unaffected is insufficient. Heat-stable proteins are ubiquitous in nature (23), and a recent study showed that abiotic materials like quartz and feldspars can also lose ice nucleation abilities when heated (24). Moreover, the UV absorbance at 280 nm, caused by aromatic residues are also present in polyketides (25), and *Fusarium* mycotoxins, which are polyketides, are enzymatically degradable (26).

Here, we investigate the composition, structure, and activity of INs from *Fusarium acuminatum,* which we extracted from the surfaces of fungal spores and mycelia. The goal of this study is to identify the nature and size of the individual ice-nucleating macromolecules and estimate how many are involved in the functional aggregates responsible for these organisms’ exceptional ice nucleation activity.

## Results

**Fig. 1.**
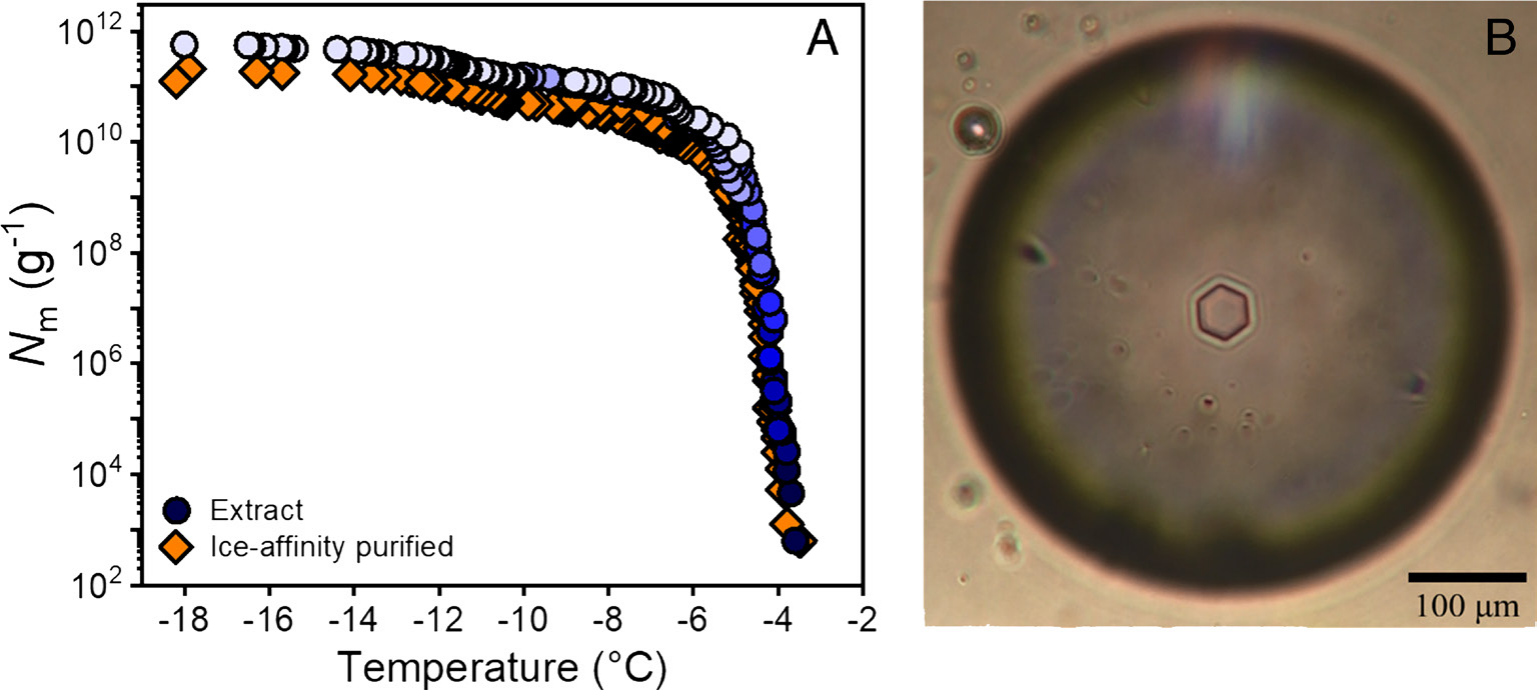
Freezing experiments of aqueous extracts containing fungal ice nucleators from *F. acuminatum*. (*A*) Shown is the cumulative number of ice nucleators per unit mass of *F. acuminatum* (*N*_m_) for extracts containing ice nucleators from spores and mycelial surfaces (blue) and for ice-affinity purified ice nucleators (orange). (*B*) Cryomicroscopic image of a hexagonal ice crystal grown in a *F. acuminatum* IN extract.

To investigate the freezing capabilities of INs from spores and mycelial surfaces of *F. acuminatum,* aqueous *Fusarium* extracts were serially diluted 10-fold, resulting in a concentration range spanning from ~14.1 mg/mL to ~1.41 ng/mL. The aqueous extracts contain all *Fusarium* INs derived from spores and mycelial surfaces and were passed through 0.1 µm filters before measurements. The freezing temperature of each of 96 3 µL-sized droplets for each concentration was followed with a cooling rate of 1 °C/min (27). These measurements provide the fraction of frozen droplets as a function of temperature for each concentration (*SI Appendix*, Fig. S1). The results are combined using Vali’s equation into a single freezing curve of *F. acuminatum,* shown in Fig. 1*A*, where *N_m_* represents the total number of active INs above a certain temperature (28). The strong increase that starts just below −4 °C, and the subsequent plateau in the cumulative number of INs per unit mass *N*_m_*(T)*, indicate the presence of highly efficient INs, consistent with previous studies (10, 21).

### The INs of *Fusarium* Are Ice-Binding Proteins.

The high ice nucleation activity in *Fusarium* is indicative of INs capable of strong binding to ice (29). We employed ice-affinity purification to capitalize on the ice-binding capabilities of the *Fusarium* INs to selectively purify them. The purification process involves the incorporation of the ice-binding INs into a slowly growing ice phase and the exclusion of non-ice-binding macromolecules and impurities. Thereby, the ice-binding macromolecules present in *F. acuminatum* were isolated. The success of the process was assessed by monitoring the activity of the purified INs and by gel electrophoresis (*SI Appendix*, Fig. S2). The freezing curve of the ice-affinity purified INs looks similar to that of the aqueous *Fusarium* extract, with a slight decrease in the total number of INs (Fig. 1*A*). The presence of ice-binding macromolecules in *Fusarium* was further investigated by measuring their ice-shaping capabilities. Using Nanoliter Cryoscopy, we observed that slow cooling of a ∼15-μm ice disc resulted in its faceting that transformed it into a hexagon, as shown in Fig. 1*B*. Measurements in the absence of INs under identical conditions (salinity, cooling rate) resulted in the formation of circular ice crystals, confirming that the purified macromolecules in the *F. acuminatum* samples selectively bind to ice.

**Fig. 2.**
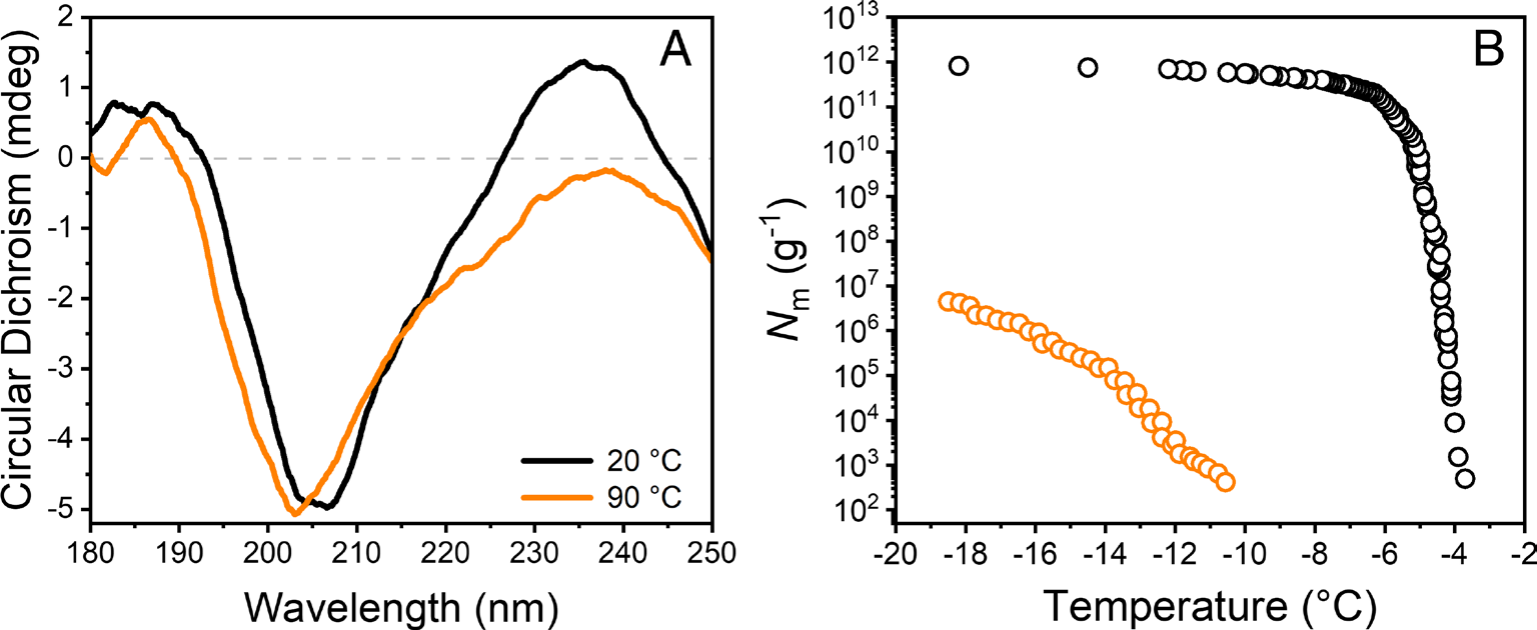
Characterization of aqueous solutions containing ice-affinity purified INs from *F. acuminatum*. (*A*) The CD spectrum shows a maximum molar ellipticity at ~235 nm and a minimum at ~205 nm, and both signals are altered following heating to ∼90 °C. (*B*) Effects of high temperature (98 °C) on the ice nucleation activity of *F. acuminatum* extract. Shown is the cumulative number of INs (*N*_m_) per gram of mycelium plotted against the temperature. Data were obtained from Kunert et al. (10).

The proteinaceous nature of the ice-binding macromolecules of *F. acuminatum* had been suggested but was not previously confirmed (10). Fig. 2*A* shows circular dichroism (CD) spectra of the ice-affinity purified solutions of *F. acuminatum* at room temperature and at 90 °C. The CD spectrum of the untreated sample shows a maximum molar ellipticity at ~235 nm and a minimum at ~205 nm. Spectral analysis and fold recognition using the web server BeStSel (30, 31) reveals that the *Fusarium* INs are proteinaceous with ~29% antiparallel β-sheet and ~12% helical content (*SI Appendix*, Fig. S3). It is worth mentioning that high β-sheet contents were also found in INPro derived from bacteria (32–36). Upon heating the ice-affinity purified *Fusarium* INs to 90 °C, the CD spectrum shows marked changes: a reduction of the molar ellipticity at 235 nm, and a shift of the minimum at ~205 nm to ~202 nm. These spectral changes following heating suggest significant, irreversible changes in the secondary structure of *Fusarium’s* INs. We interpret that these conformational changes cause an irreversible loss of the protein’s native structure and are the origin of the significant loss of *Fusarium*’s ice nucleation activity after heat treatment (Fig. 2*B*) (10).

### Determination of IN Subpopulations Based on Numerical Modeling and Nucleation Theory.

**Fig. 3.**
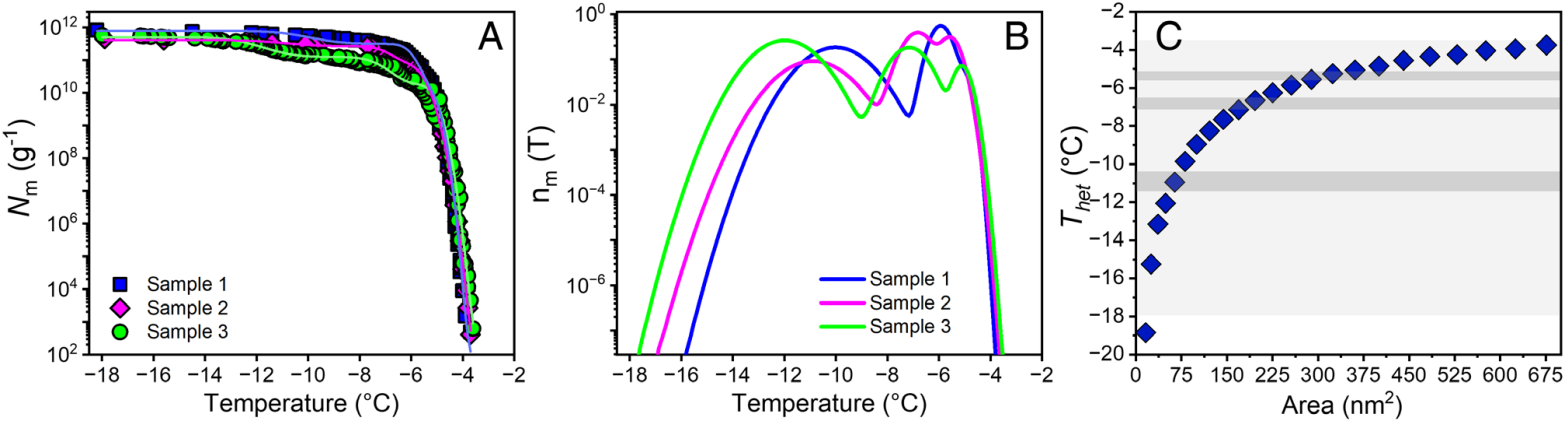
Freezing experiments of aqueous extracts containing fungal INs from *F. acuminatum*. (*A*) Cumulative number of INs per unit mass of *F. acuminatum* (*N_m_*) for extracts containing INs from spores and mycelial surfaces. The lines represent the optimized solution obtained through the HUB-backward code assuming that the differential spectrum is a combination of three Gaussian subpopulations. The colors of the lines are the same as for the sets they fit. (*B*) Normalized distribution function that represents the corresponding differential freezing spectrum $n_{m}(T$ ). The distributions have modes around −5.5, −6.8, and −11 °C (*SI Appendix*, Table S1); however, their overlap and comparable proportions support that there is a continuum of IN sizes. (*C*) Ice nucleation temperatures as a function of ice-binding area for the INs from *F. acuminatum*. Blue diamonds show the freezing temperatures of square surfaces predicted by classical nucleation theory(CNT) implemented in the HINT algorithm. The light gray shaded area represents the full range of the heterogeneous freezing temperatures, and the middle of the dark gray shaded areas represents the modes of the populations in the differential spectrum. The range of temperatures were determined from the SD of each Gaussian distribution.

Fig. 3*A* presents *N*_m_(*T*) obtained from dilution series of three independent experiments with aqueous extracts from individual fungal cultures. To obtain the distribution of heterogeneous ice nucleation temperatures of the fungal INs, we extracted the differential freezing spectrum *n*_m_(T) from each experimental cumulative freezing spectrum *N*_m_(*T*) using the heterogeneous underlying-based (HUB) backward code (37). The code implements a stochastic optimization procedure that enables the fitting of the experimental *N*_m_(*T*) data, as shown in Fig. 3*A*, assuming that the distribution of nucleation temperatures in the *Fusarium* sample is a linear combination of Gaussian subpopulations. The mean square error (MSE) between the experimental and predicted *N*_m_(*T*) decreases from ~15 to 1% as the number of subpopulations increases from one to three (*SI Appendix*, Fig. S4 and Table S1). The differential spectra *n*_m_(T) (Fig. 3*B*) that best fits the experimental *N*_m_ (*T*) of Fig. 3*A* contain three Gaussians centered around −5.5, −6.8, and −11 °C (*SI Appendix*, Table S1). The comparable contribution and overlap of these subpopulations imply an almost continuum distribution of IN sizes. This suggests that the subpopulation of more potent IN dominates the freezing of water by the organism in its natural environment.

*SI Appendix*, Fig. S8 shows that consecutive freeze–thaw cycles of the same sample have only minor impacts on the ice nucleation ability of IN from *F. acuminatum* and the corresponding freezing spectrum. In contrast to fungal INs, repetitive freeze–thaw cycles alter the freezing spectra of bacterial INs, and the activity of their most active INs is significantly reduced (38). The known instability of bacterial INs (39), is in sharp contrast to the observed high stability of fungal INs (10).

To interpret the position of the peaks in the differential spectrum $n_{m}\left(T\right)$   of *F. acuminatum*, we use an accurate implementation of CNT to predict ice nucleation temperatures of finite-sized IN surfaces (29). The calculation is implemented into the “Heterogeneous Ice Nucleation Temperature” (HINT) code, which takes into account the size and shape of the surface, and temperature-dependent thermodynamic and dynamic water properties to predict the temperature of heterogeneous ice nucleation (29). We first assume that the ice-binding area of the fungal IN is a flat square surface with a binding energy *Δ*γ corresponding to that of ice binding to ice. These calculations provide a lower limit for the area of the IN, because a less potent *Δ*γ or an elongated IN would require a larger area to reach the same nucleation temperature (29). The predicted freezing temperatures based on square IN surfaces are shown in Fig. 3*C*. The ice nucleation signal for the three modes of the differential spectrum $n_{m}\left(T\right)$ of *F. acuminatum* match those of square IN surfaces of areas 290 nm^2^ (*T*_het_ = −5.5 °C), 187 nm^2^ (*T*_het_ = −6.8 °C), and 53 nm^2^ (*T*_het_ = −11 °C) (Fig. 3*C* and *SI Appendix*, Table S2). We emphasize that these areas are the minimal ones required to nucleate ice at the corresponding temperatures, and match those of squares with sides 17, 13.7, and 7.3 nm, respectively. For reference, the narrower rectangular surface that nucleates ice at −6.8 Å has a width of 8.4 nm and a length of ~45 nm, resulting in about twice the area of the 13.7 × 13.7 nm^2^ surface that promotes ice nucleation at that temperature. Below we use the estimated square IN areas to provide lower bounds of the number of protein monomers involved in the ice-nucleating particles produced by *F. acuminatum.*

Our theoretical estimate of the size of the IN surfaces responsible for the exceptional nucleation activity of *F. acuminatum* is consistent with the results of filtration experiments that showed *N*_m_ to be unchanged by filters with nominal cutoff down to 100 nm, minimally impacted by filters with nominal cutoff at ~9 nm (300 kDa) and strongly impacted by filters with smaller pores (*SI Appendix*, Fig. S5) (10, 21). However, neither the filtration experiments nor our theoretical calculations can reveal whether the INs are composed of smaller subunits, and, if they are, what is their actual size.

### Determination of Size and Composition of *Fusarium* INs.

**Fig. 4.**
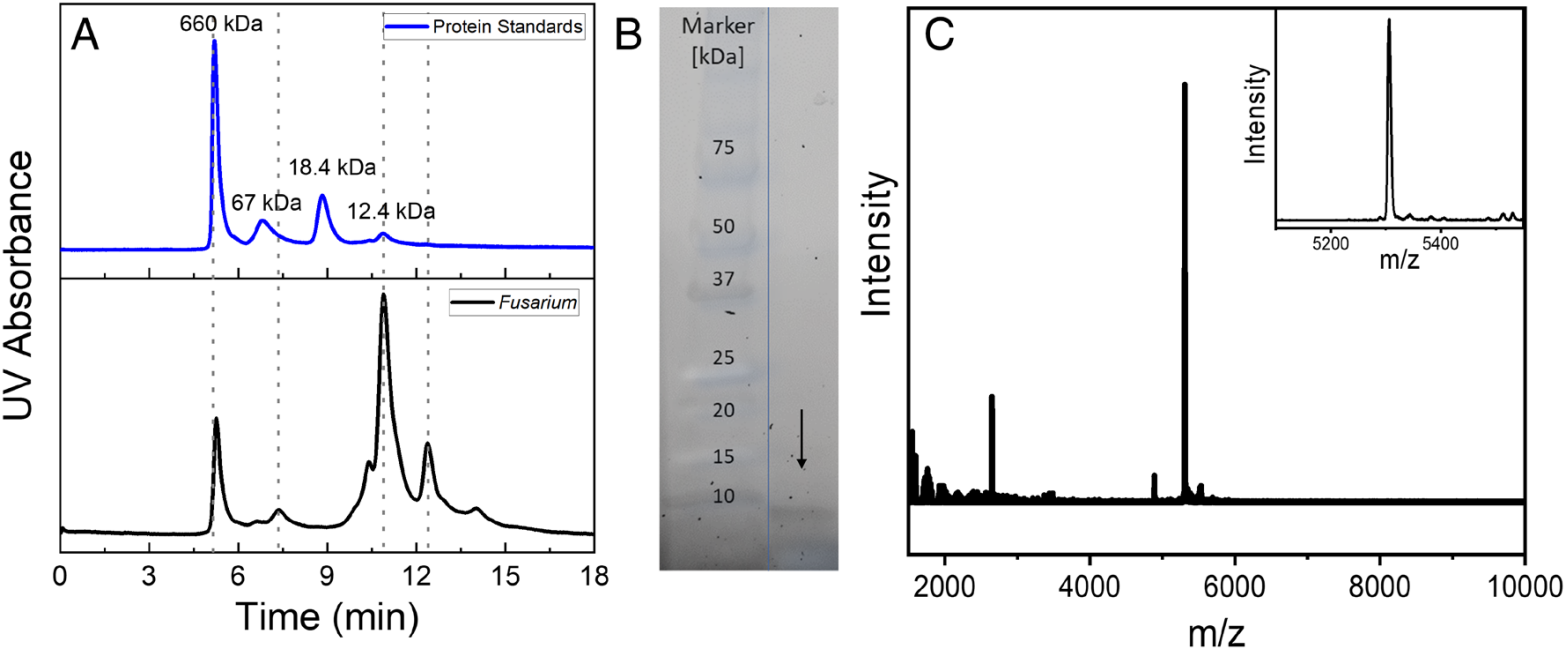
Characterization of aqueous solutions of ice-affinity purified INs from *F. acuminatum*. (*A*) Separation of a protein standard and the ice-affinity purified *F. acuminatum* extract on a gel filtration column using a TSK G2000SWXL. The proteins were eluted with a 50 mM sodium phosphate, 0.3 M NaCl buffer, pH 7, at a flow rate of 1 mL/min. (*B*) SDS-PAGE gel electrophoresis of the ice-affinity purified *F. acuminatum* extract shows a weak band just below 10 kDa that is highlighted by an arrow. (*C*) MALDI spectra of the ice-affinity purified sample show a dominant signal at ~5,300, which is highlighted in the inset.

To address these questions, we used size exclusion chromatography (SEC), gel electrophoresis, and matrix-assisted laser desorption/ionization-time-of-flight (MALDI-TOF) mass spectrometry of the ice-affinity purified *Fusarium* INs. Fig. 4*A* shows the SEC separation profile of standard calibration proteins and *Fusarium* INs in 50 mM sodium phosphate in 0.3 M NaCl buffer (pH 7.0). The elution profile of the *Fusarium* samples showed prominent peaks at ~5.20, ~7.40, ~10.98, and ~12.40 min, and a smaller peak at ~13.5 min (Fig. 4*C*). Based on the elution profile of the calibration proteins, we estimate the molecular weight of the *Fusarium* elution peaks to be ~660, ~45, ~12, ~6, and ~ <3 kDa, respectively. Given that proteins can vary significantly in shape, the determined molecular weights of the *Fusarium* INs should not be seen as absolutes, but rather as good approximations. Furthermore, the column’s reduced sensitivity towards high kDa proteins may cause large aggregates to appear similar in size. The observed “660 kDa” peak could act as a cut-off point for larger aggregates and include a range of larger sizes.

We find that both large (~660 kDa) and small (~12 kDa) SEC *Fusarium* fractions retain their ice nucleation activity at low supercooling (*SI Appendix*, Fig. S6 and Table S1). The IN peaks for the 660 kDa fraction (−7.5, −6.1, and −4.7 °C) overlap with the two warmer IN peaks obtained from the whole sample (−6.8 and −5.3 °C), while clearly lacking the population of less active nucleants (around −11 °C) obtained from the whole *Fusarium* extract. On the other hand, the three IN peaks obtained from the 12 kDa sample (−11.8, −8.5, and −6.4 °C) encompass those found in the two less efficient peaks of the whole sample, while lacking the most potent IN population around −5 °C. These results strongly suggest that even when small subunits are initially present, there exists a robust mechanism for them to reassemble into larger INs. Without a reassembly mechanism, the experimentally observed activity of the ~12 kDA SEC fraction at higher temperatures would not be possible (Fig. 3*C*).

The presence of small subunits with an estimated weight below 10 kDa is supported by MALDI spectra of ice-affinity purified solutions of *F. acuminatum* and SDS-PAGE gel electrophoresis (Fig. 4*B*). The MALDI spectrum shows a dominant signal at ~5.3 kDa, with a second smaller signal at 2.65 kDa, which either originates from a z = +2 charge state of the 5.3 kDa protein, or a separate half-sized protein. Considering the 110 Da average molecular weight of amino acids, we estimate that the ~5.3 kDa protein unit contains ~48 amino acids. Interestingly, the presence of a peak at 2.65 kDa, which is half the size of the dominant peak at 5.3 kDa, along with a minor fraction in the SEC profile, raises the possibility that the 5.3 kDa protein might be a dimer of a smaller subunit. Furthermore, the faint second band observed in the SDS PAGE hints at the potential existence of a smaller component, although it could also be an artifact from the dye front. The SDS-PAGE gel shows a band below ~10 kDa, confirming the presence of small proteins. We further performed an amino acid analysis of the ~12 kDa SEC fraction to verify the proteinaceous nature of the *Fusarium* IN. The amino acid content showed high contents of aspartic (15%) and glutamic acids (19%) (acid or amide form) as well as of threonine (10%) and serine (8%) (*SI Appendix*, Fig. S7). A globular protein of ~10 kDa would have a molecular diameter of ~2.8 nm (40) and nucleate ice at temperatures below −23 °C (*SI Appendix*, Table S2). The much warmer nucleation temperatures obtained with the 12 kDa SEC fraction (*SI Appendix*, Fig. S6 and Table S1) evince that *Fusarium* INs consist of small protein units that assemble into larger complexes in solution.

We assessed the minimum number of protein monomers needed to achieve the nucleation temperatures of *F. acuminatum* through a combination of experiments, nucleation theory, and modeling results. If we assume that the 5.3 kDa monomer is the smallest unit and it has a globular fold, then its radius would be ~1.1 nm (40) and would nucleate ice at about −28 °C (*SI Appendix*, Table S2). A functional assembly of at least 150 5.3 kDa units would be required to tile the minimal IN surface that nucleates ice at −4 °C, ~100 to nucleate at −5 °C, ~50 at −6.8 °C, and ~16 at −11 °C (*SI Appendix*, Table S2). This range of aggregate sizes is consistent with those observed in the size exclusion chromatography of the ice-affinity purified solutions (Fig. 4*A*).

## Discussion

We combine physicochemical characterizations of ice-affinity purified *Fusarium* INs with stochastic optimization algorithms and nucleation theory to unravel the nature and size of the units and functional assemblies that endow this fungus with outstanding ice nucleation abilities. We find that the fungal INs consist of small protein subunits with about 50 amino acids that assemble in a cell-free environment into functional aggregates that enable ice nucleation at low supercooling. These functional aggregates can contain over a hundred ice nucleating proteins. Both experimental and theoretical evidence indicate the existence of functional aggregates with a continuum distribution of sizes, highlighting the need for further investigations that elucidate their structures and the mechanisms governing their assembly. Notably, the *Fusarium* INs can regain ice nucleation activity at low supercooling even when only the SEC fraction corresponding to small <12 kDa proteins are initially present (*SI Appendix*, Fig. S6), suggesting the existence of robust mechanisms for their efficient reassembly in cell-free aqueous environments.

In bacteria, it has been shown that dimers and higher-order oligomers are key for the activity of ~120 kDa INPro (4, 19, 35, 36). *P. syringae* display functional INPro aggregates in their outer membrane that can nucleate ice at high onset temperatures (4, 19, 29, 35, 36). However, individual bacterial INPro nucleate ice at just −16 °C, and while aggregation of bacterial INPro probably occurs in solution (36), the membrane is needed to produce the most active form (41). The ability to regain high ice nucleation activity from monomers in solution and their high stability sets *Fusarium* INPro apart from bacterial ones, which lack these capabilities (29, 35, 41, 42). Here, we demonstrate that functional aggregation of much smaller ~5.3 kDa proteins from fungi also results in large IN assemblies. Likewise, control of ice nucleation by assembling large units is also common in insects and pollen, where a combination of carbohydrates, lipids, and proteins enables freezing (43, 44). We conclude that nature has implemented these *E pluribus unum* strategies across biological kingdoms using a wide range of sizes of protein building blocks, from ~120 kDa in bacteria to ~5 kDa in fungi.

The functional aggregation processes associated with these strategies give rise to potent ice-nucleating structures, which can manifest as soluble extracellular aggregates in fungi or membrane-bound assemblies in bacteria. In all cases, however, the functional assembly of smaller units results in a sufficiently large ice-binding area that can support the formation of critical ice nuclei at low supercooling (29, 45). We expect that the energetic benefit for the organism in producing smaller proteins, rather than a single large one, contributes to the success and adoption of the *E pluribus unum* strategy across species that are not evolutionary-related. The same strategies could be applied to design and produce synthetic INs by self-assembly of small ice-binding structures. Developing such powerful synthetic INs would be highly valuable for applications ranging from cryopreservation of cells to cloud seeding.

## Methods

### Fungal Culture and Sample Preparation.

Seventy-five plates of the ice nucleation-active fungal species *Fusarium acuminatum* were grown on full-strength potato dextrose agar plates (VWR International GmbH). Growth occurred at room temperature for one week and then at 6 °C for about four weeks. Pure water was obtained from Millipore Milli-Q® Integral 3 water purification system (Merck Chemicals GmbH), autoclaved at 121 °C for 15 min, and filtered through a 0.1 µm bottle top filtration unit (VWR International GmbH). For the droplet freezing experiments, aqueous extracts of fungal mycelium were prepared as described previously with the following modifications (10). The fungal mycelium of five agar plates was collected in a sterile 50 mL tube, and the weight of the mycelium was determined gravimetrically. Aliquots of 50 mL of pure water were added to the mycelium. The samples were vortexed three times at 2,700 rpm for 1 min. The aqueous extracts for all experiments were filtered through a 0.1 µm bottle-top filtration unit (VWR International GmbH), and the resulting aqueous extracts contained ice nucleators from spores and mycelial surfaces. For filtration experiments, the 0.1 μm filtrate was filtered through either 30 kDa or 50 kDa MWCO PES ultrafiltration centrifugation units (Thermo Fisher Scientific), and the ice nucleator concentration was determined by TINA measurements.

### Ice-Affinity Purification.

Rotary ice-shell and ice-slide purification was used to purify the ice-nucleating macromolecules of the crude fungal extract. Details of the purification method have been described elsewhere (32, 46). In short, in a 500 mL flask, ~20 to 30 mL ultrapure water was used to form an ice-shell using a dry ice-ethanol bath for 30 to 60 s. The flask was then rotated in a temperature-controlled ethylene glycol bath, and the temperature of the bath was set to −2 °C. Then, 100 mL precooled fungal extract was added, and the flask rotated continuously in the bath until 30% of the solution was frozen. The ice was melted and freeze-dried to obtain a mixture of the ice-binding macromolecules present in *F. acuminatum*. The ice-affinity purification procedure is typically performed multiple times (2–5 times), and the success of the purification was checked by determining the ice nucleation activity of the ice-affinity purified *Fusarium* samples using TINA measurements and by gel electrophoresis (*SI Appendix*, Fig. S1). The ice-affinity purified solution was used for SEC experiments and to obtain MALDI, SDS-PAGE, and CD spectra.

#### Size Exclusion Chromatography.

The ice-affinity purified extract was lyophilized and dissolved in water. The protein in the clear solution (~4 mg/mL) was analyzed by HPLC using a G2000SWXL TSK gel column (7.6 mm × 30 cm). The elution buffer was 0.05 M sodium phosphate in 0.3 M NaCl, pH 7.0. The flow rate was 1 mL/min, with absorbance recorded at 220 or 280 nm.

#### SDS-PAGE.

Aliquots of the ice-affinity purified extracts were mixed with a fifth volume of 6x Laemmli buffer containing 5% of β-Mercaptoethanol and were heated at 95 °C for 5 min. Samples were loaded onto a MiniPROTEAN® TGX™ Stain-free Precast Protein Gel (4–20%, Bio-Rad) next to a molecular weight marker (Precision Plus Protein Unstained Standards, 161-0363, Bio-Rad). The electrophoresis setting was a constant voltage of 175 V for 40 min. Image acquisition of the gel was performed using a ChemiDoc MP Imaging system and the Image Lab software (Version 5.1, Bio-Rad).

### TINA Experiments.

Ice nucleation experiments were performed using the high-throughput Twin-plate Ice Nucleation Assay (TINA), which has been described in detail elsewhere (27). In a typical experiment, the investigated IN sample was serially diluted 10-fold by a liquid handling station (epMotion ep5073, Eppendorf). Then, 96 droplets (3 µL) per dilution were placed on two 384-well plates and tested with a continuous cooling rate of 1 °C/min from 0 °C to −30 °C with a temperature uncertainty of ±0.2 °C. The droplet-freezing was determined by two infrared cameras (Seek Therman Compact XR, Seek Thermal Inc.). For each experiment, the obtained fraction of frozen droplets (*f*_ice_) and the counting error were used to calculate the cumulative number of IN (*N*_m_) with the associated error using Vali’s formula and Gaussian error propagation (28). All experiments were performed multiple times (e.g., Fig. 1, 3–5 samples). We find that independent samples from individual fungal cultures show similar results with some variations, consistent with a previous study (10).

### CD Spectroscopy.

CD spectra of the ice-affinity purified extracts were recorded at a 1 nm interval from 260 to 180 nm using a Jasco J-1500 spectrometer. CD measurements were performed in a rectangular cell with the optical path of 0.1 cm. CD measurements were taken at 90 °C or room temperature, and the equilibration time for every sample before each set of measurements was 15 min. All spectra were background subtracted and processed using the Spectra Manager Analysis program from JASCO.

### MALDI-TOF.

MALDI measurements were carried out on a rapifleXTM MALDI-TOF/TOF mass spectrometer from Bruker Daltonik GmbH. The instrument is equipped with a scanning smartbeam 3D 10 kHz Nd:YAG laser at a wavelength of 355 nm and a 10-bit 5 GHz digitizer. The acceleration voltage was set to 20 kV and the mass spectra were recorded in positive ion mode. Calibration was done with the Bruker peptide mix and the Bruker protein calibration standard I and II in a mass range of up to 70 kDa. Sample preparation was done by mixing a saturated solution of sinapinic acid dissolved in water/acetonitrile (1:1 + 0,1% trifluoroacetic acid) with an aqueous solution of the analyte in equal amounts. Aliquots of the ice-affinity purified extracts were measured with random walk ionization across the sample spot. Typically, 8,000 shots were averaged per spectrum.

### Nanoliter Cryoscopy.

Ice shaping was determined at a *Fusarium* extract concentration of ~10 mg/mL in water using a Clifton Nanoliter Osmometer (47). Ice shaping was performed with a cooling rate of 0.075 °C/min and without annealing. Measurements were performed 2–4 times on independent samples.

### HUB Method.

We used the HUB-backward stochastic optimization code to extract the distribution of heterogeneous ice nucleation temperatures from the experimental cumulative nucleation spectra (37). The HUB code uses the same assumptions adopted by Vali (28). It considers that the number of IN in each droplet follows the Poisson distribution, that each IN has a distinct nucleation temperature, and that the IN with the warmest nucleation temperature in the droplet sets the freezing temperature of the droplet in the cooling experiment. The HUB-backward code represents the distribution of nucleation temperatures of the IN in the sample as a linear combination of Gaussian populations and uses a stochastic optimization procedure to find the best set of parameters of the population -modes, widths, and weights- to reproduce the experimental *N*_m_(*T*). The output of the HUB-backward is the differential spectrum *n*_m_(*T*) in terms of the distribution of subpopulations of INs that reproduce the cumulative freezing spectrum *N*_m_(*T*) of *Fusarium*.

### CNT.

The HINT algorithm is an accurate numerical implementation of CNT that predicts the temperatures of heterogeneous nucleation of ice on finite-sized IN surface using experimental data for water such as the self-diffusion coefficient *D*, the difference in chemical potential between liquid and ice ∆*μ*, and the ice–liquid surface tension *γ*_ice-liquid_ (29), the surface binding free energy of the IN to ice ∆γ_bind =_ *γ*_ice-IN_ − *γ*_ice-liquid_ − *γ*_liquid-IN_, where *γ*_ice-IN_ and *γ*_liquid-IN_ are the surface tensions of the ice nucleating surface with ice and liquid water, respectively (29). HINT uses that data to compute the free energy barriers for ice nucleation and the prefactor for the nucleation rate. It predicts the nucleation temperature with that data and knowledge of the experimental nucleation rate *J*_exp_ = 10^5^ cm^−3^ s^−1^ corresponding to cooling microliter at rates of 1 °C/min (48–50). We assume that the IN binds ice as strong as ice itself, i.e., ∆γbind = −2 *γ*_ice-liquid_, as deduced for the ice nucleating proteins of *P. syringae* (29). Any weaker binding would require larger IN surfaces to reach the same ice nucleating temperature. We further assume that the IN surface is a square, because this compact shape requires lower area to nucleate ice at a given temperature than any other rectangular shape. We follow the procedures of Qiu et al. (29) to compute with HINT the minimum width of an IN that nucleates ice at −6.8 °C, 8.4 nm, by first assuming that the IN is an extremely long (80 nm long) of width W, adjusting W down from the 13.7 nm value of the square until we find that the ice nucleation occurs at the target −6.8 °C, and then shortening the length in 5 nm decrements until the freezing temperature falls below −6.8 °C. That results in an estimate of 45 nm for the minimum length of the narrower aggregate that can nucleate ice at −6.8 °C. As the smaller IN area corresponds to a square shape, we use the results for the square surfaces as a lower bound for the true areas of the IN aggregates of *Fusarium*.

## Supplementary Material

Appendix 01 (PDF)

## Data Availability

All study data are included in the article and/or *SI Appendix*.
