# Supplementary material for: Functional aggregation of cell-free proteins enables fungal ice nucleation
Source: Proc Natl Acad Sci U S A. 2023 Nov 9;120(46):e2303243120. doi: 10.1073/pnas.2303243120 (PMC10655213; doi:10.1073/pnas.2303243120)
Supplement: Supplementary file 1 — Appendix 01 (PDF) [file pnas.2303243120.sapp.pdf]

Supporting Information

Title: Functional Aggregation of Cell-Free Proteins Enables Fungal Ice Nucleation

Authors: Ralph Schwidetzky[a,1], Ingrid de Almeida Ribeiro[b,1], Nadine Bothen[c], Anna T. Backes[c], Arthur L. DeVries[d], Mischa Bonn[a], Janine Fröhlich-Nowoisky[c], Valeria Molinero[b,2], and Konrad Meister[a,e,2]

Affiliations:

[a]Max Planck Institute for Polymer Research, 55128 Mainz, Germany

[b]The University of Utah, 84112 Salt Lake City, UT, United States

[c]Max Planck Institute for Chemistry, 55128 Mainz, Germany

[d]University of Illinois at Urbana-Champaign, 61801 Urbana, IL, United States

[e]Boise State University, 83725 Boise, ID, United States

Filtration Experiments

Fig. S5 shows freezing curves of *F. acuminatum* IN solutions after subsequently passing them through 50 and 30 kDa molecular weight cut-off (MWCO) filters. Filtrations decreased the cumulative number of INs per gram of mycelium and shifted the initial freezing temperature towards lower temperatures. As shown in Fig. S5A, a very significant ice nucleation activity remained ($N_m(T)$ ~−5 °C) even after passing through a 30 kDa filter. By comparing the N_m plots and the fraction of ice of the filtered and unfiltered solutions (Fig. S5B), we find that the number of *Fusarium* INs after passing through a 50 kDa filter, appears similar to the 10^{-5} dilution of the initial sample. The similarity of filtered and diluted samples is further supported by dynamic light scattering experiments that show comparable hydrodynamic radii of ~200 nm for different dilutions of the *Fusarium* extracts and filtered samples (Fig. S5C). The finding that ice nucleation activity of *Fusarium* INs persists in the filtrates provides additional evidence that the aqueous extracts of *Fusarium* INs consist of smaller units which aggregate to larger ice-nucleating complexes in solution. However, it is important to note, that the molecular weight cut-off (MWCO) in filters are nominal classifications and not precise boundaries (1). Typical filters contain a broad range of pore sizes, making it impossible to achieve 100% retention of even very large molecules, and thus will detort conclusions drawn solely based on them.

Estimation of Protein Subunits in the Aggregates

We estimate the area of the 5.3 kDa protein assuming, for simplicity, that is has a globular fold. The approximate radius of a 5 kDa protein is 1.1 nm (2), resulting in a projected area $A_m = \pi * 1.1^2\ nm^2 = 3.8\ nm^2$ for the monomer. The number of the required subunits that enable freezing is then calculated by dividing the value obtained through the HUB and HINT analysis by A_m. This calculation assumes that the IN assembly consists of a single well-packed layer of the ice nucleating monomers. As such, and considering that we assumed that the IN are as good as ice at promoting ice nucleation and that the IN surfaces are square, the values reported are a lower bound for the actual number of monomers in the IN aggregates of *F. acuminatum*.

Dynamic Light Scattering

Dynamic light scattering (DLS) measurements were performed on an ALV spectrometer consisting of a goniometer and an ALV-5004 multiple-tau full-digital correlator (320 channels), which allows measurements over an angular range from 30° to 150°. A He-Ne Laser (wavelength of 632.8 nm) was used as the light source. Measurements were performed at 20 °C at 9 angles ranging from 30° to 150°. The hydrodynamic radii (R_h) of ~1 mg/mL filtered *F. acuminatum* solutions were determined using DLS. The R_h of the smaller and larger fractions of *F. acuminatum* was found to be similar.

Amino Acid Analysis.

Amino acid analysis was performed by the Molecular Structure Facility at UC Davies as described elsewhere (3) and the used samples were ice-affinity purified and filtered.

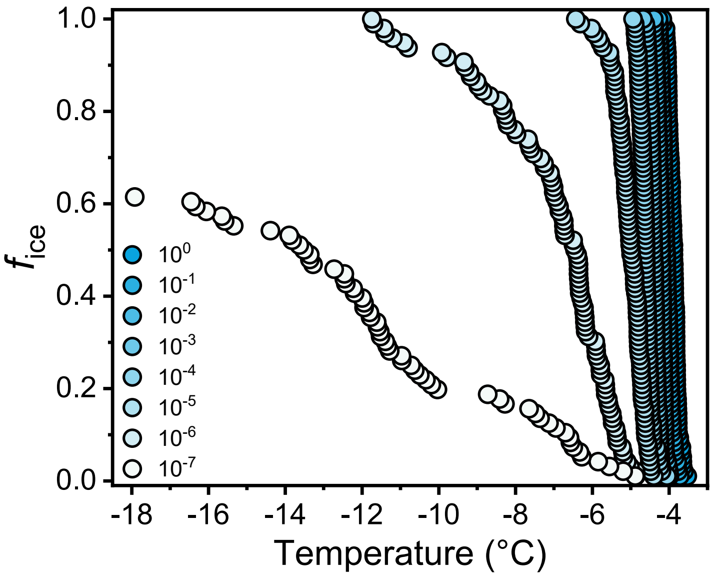

Fig. S1. Fraction of ice (f_{ice}) for different dilutions of aqueous extracts containing INs from spores and mycelial surfaces from *F. acuminatum*. The presented data corresponds to the N_m plot shown in Fig. 1A of the main text.

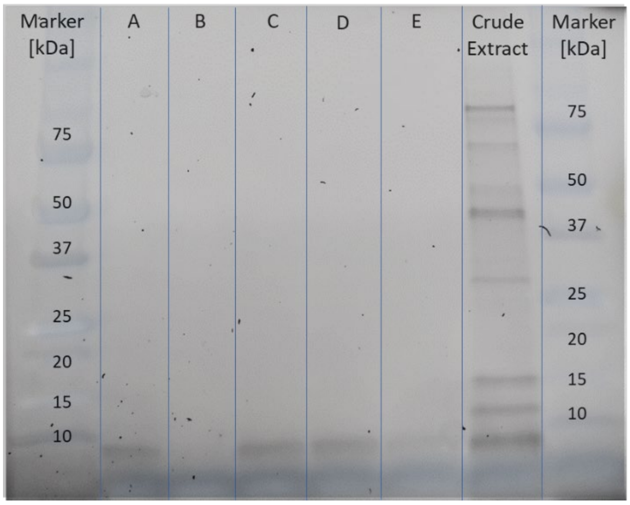

Fig. S2. SDS PAGE gel of the crude *Fusarium* extract, ice-purified INPro and molecular weight markers. Lanes A-E show different purified and filtered *Fusarium* IN samples. The impurities that are visible in the crude extract were removed in the purified fractions and a band at <~10 kDa remains.

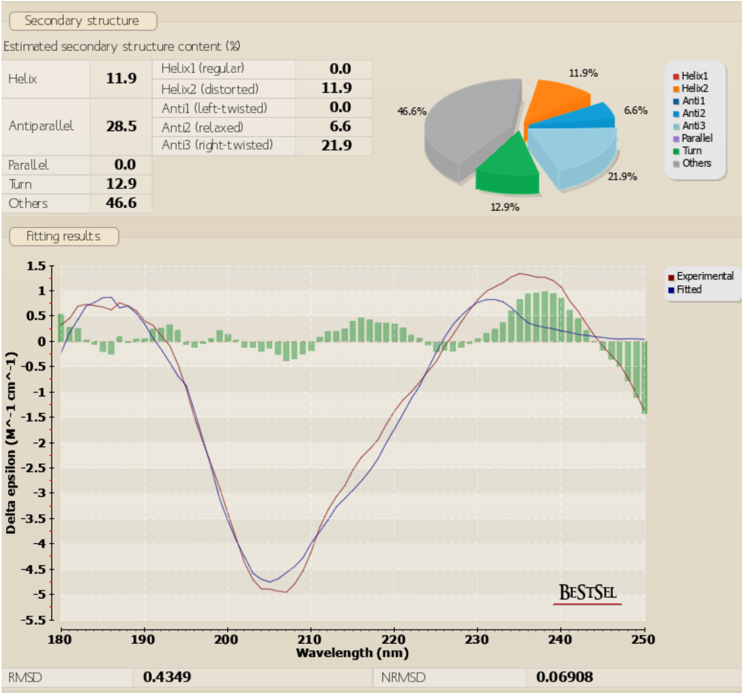

Fig. S3. Secondary structure contents of the *Fusarium* INPro as determined by BeStSel (3, 4). BeStSel is a webserver for protein secondary structure prediction and fold recognition from circular dichroism spectra. The analysis shows that the *Fusarium* INPro have a ~29% antiparallel β-sheet and a ~12% helical content.

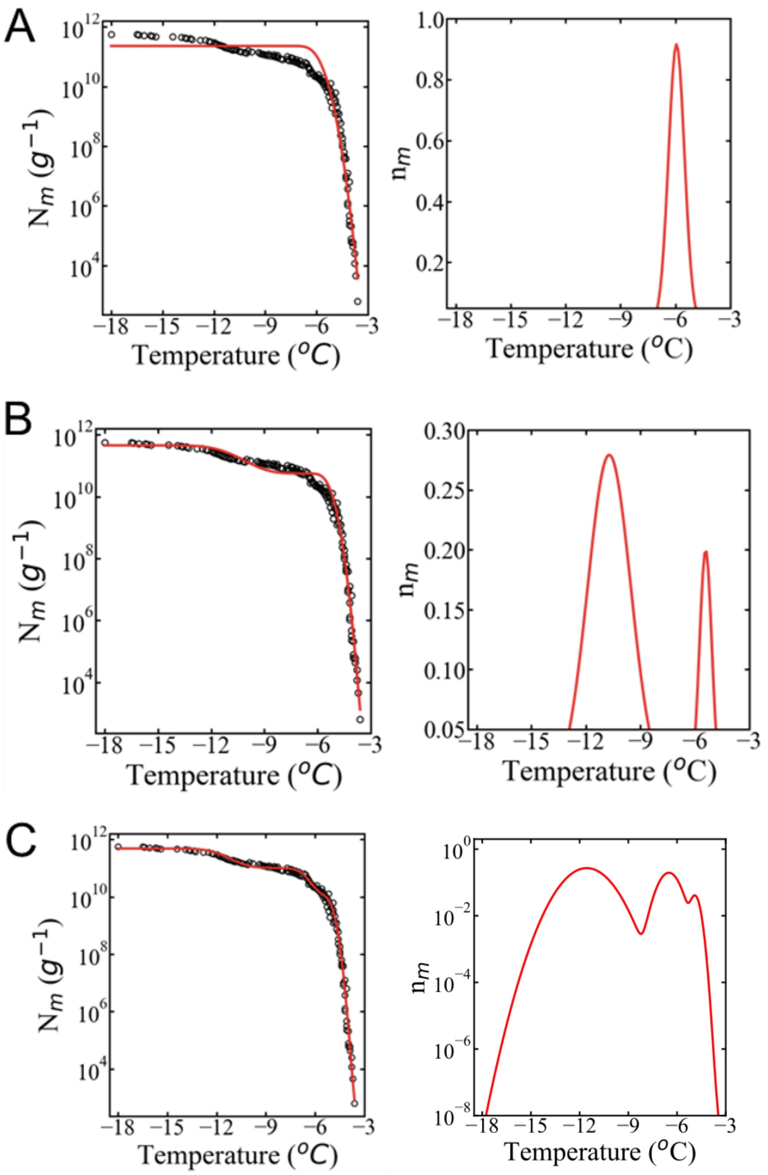

Fig. S4. Cumulative number of INs per unit mass of *F. acuminatum* (N_m) for extracts containing INs from spores and mycelial surfaces. The red line represents the optimized differential spectrum obtained through the HUB-backward code assuming that it is a combination of one (A) two (B) or three (C) Gaussian subpopulations. Normalized distribution function that represents the corresponding differential freezing spectrum n_m(T). The mean square error (MSE) between the experimental and predicted $N_m(T)$ decreases from 14.4% to 3.4% to 1.1% as the number of subpopulations increase from one to three.

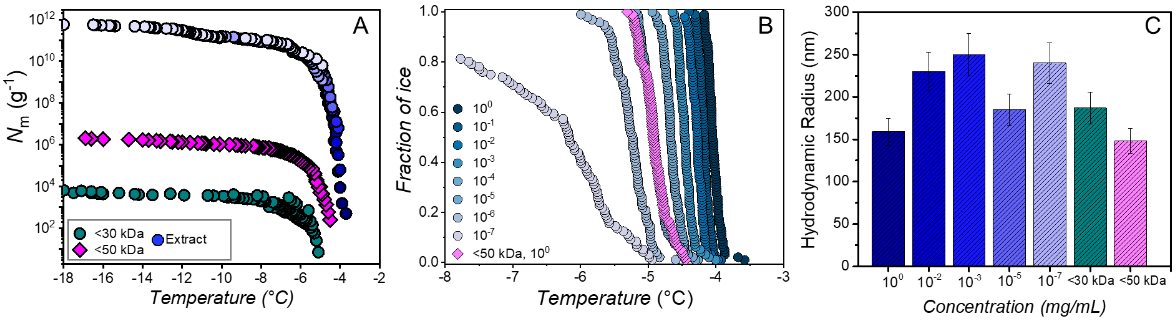

Fig. S5. Size determination of fungal ice nucleators (INs) from *F. acuminatum* upon filtration (A) Cumulative number of INs per unit mass of *F. acuminatum* (N_m) for extracts containing INs from spores and mycelial surfaces and samples that were passed through 50 kDa (magenta diamonds) and 30 kDa (green circles) filters. (B) Fraction of ice for different dilutions of INs from *F. acuminatum* and the highest concentration of the sample that was passed through a 50 kDa filter. (C) Hydrodynamic radii for different dilutions of fungal INs and of samples passed through 50 kDa (magenta) and 30 kDa (green) filters. Radii were determined using dynamic light scattering and error bars represent the standard error of the measurements.

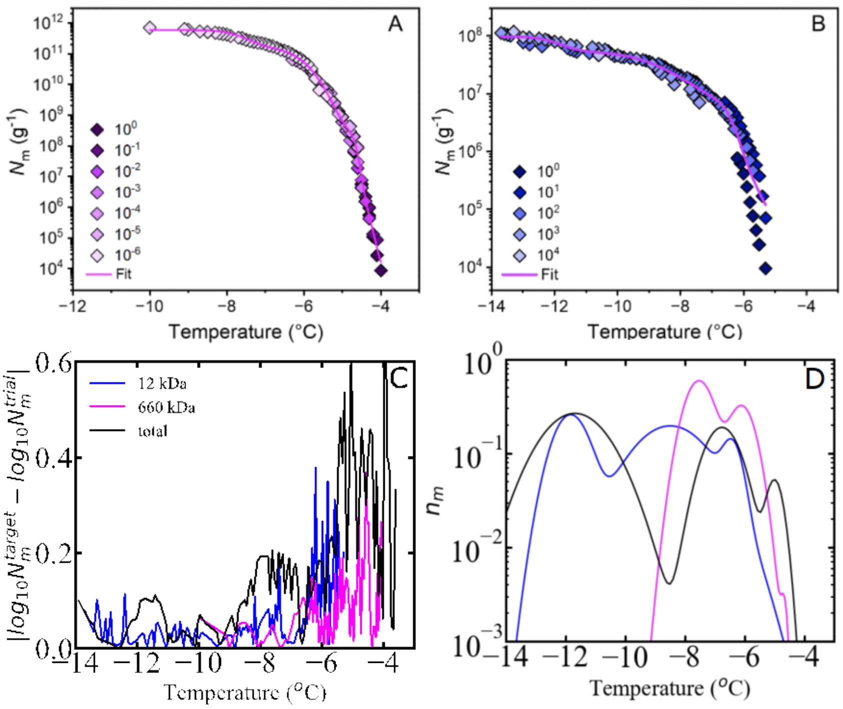

Fig. S6. Freezing experiments of aqueous extracts containing fungal INs from *F. acuminatum*. (A) Cumulative number of INs per unit mass of *F. acuminatum* (N_m) for the largest (~660 kDa) SEC fraction. (B) Cumulative number of INs per unit mass of *F. acuminatum* (N_m) for the smallest (~12 kDa) SEC fraction. We estimate the concentrations of the SEC fractions to be ~1.6 mg/mL for the larger and 0.3 mg/mL for the smaller fraction. The differential spectrum n_m(T) that best fits the smaller fraction has three subpopulations. The modes of the distributions are -6.4, -8.5, and -11.8 °C for the small (~12 kDa) SEC fraction and the mean square error (MSE) between the experimental and predicted $N_m(T)$ is 1%. (C) The difference of the logarithm of N_m^{target} and the logarithm of N_m^{trial} as a function of temperature. (D) The differential freezing spectrum with three subpopulations estimated using the HUB-backward code.

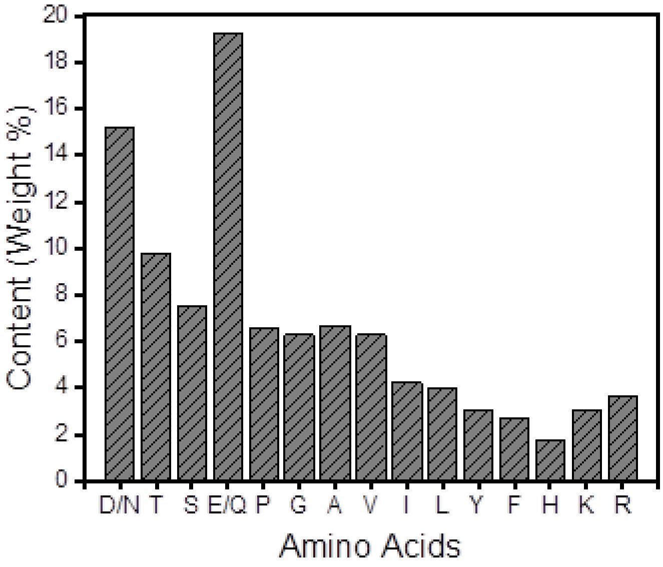

Fig. S7 Amino acid composition of the small (<12 kDA) SEC fraction of the ice-purified INPs from *F. acuminatum* in weight %. The amino acids aspartic (15%) and glutamic acids (19%) (acid or amide form) as well as threonine (10%) and serine (8%) show the highest contents.

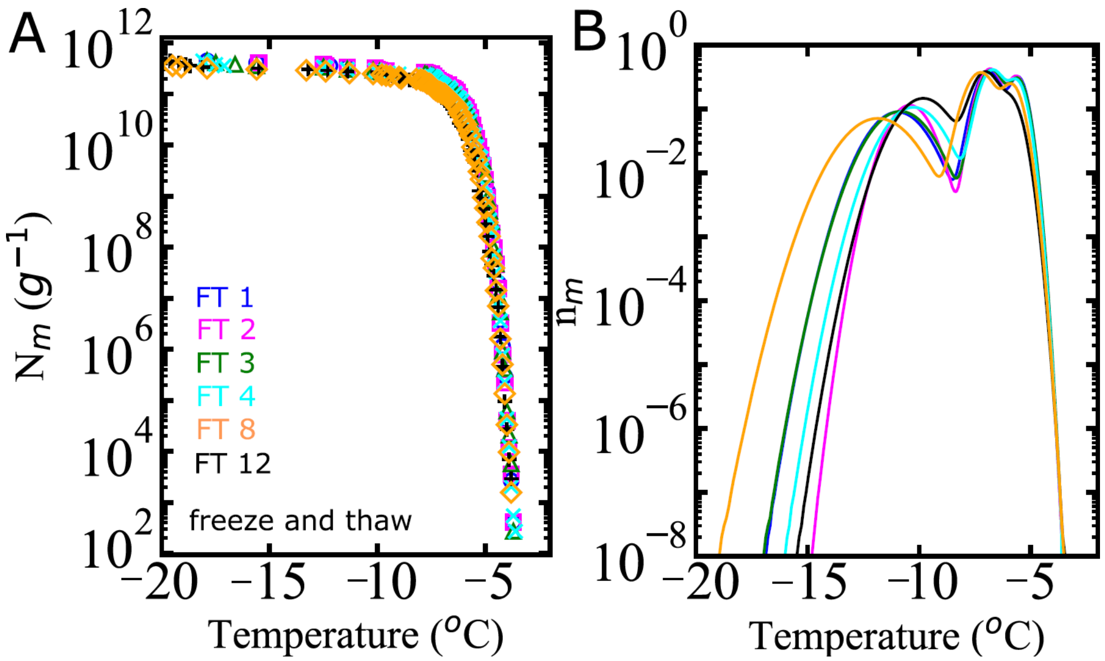

Fig. S8. Impact of freeze-thaw cycles and sampling on the activity of IN from *F. acuminatum.* (A) Cumulative number of INs per unit mass of *F. acuminatum* (N_m) for the same sample after twelve freeze-thaw (FT) cycles. (B) Differential spectra (n_m) corresponding to the distribution of ice nucleating temperatures of the IN along these FT cycles. The modes, spread, and weights of the subpopulations of these FT-treated samples are shown in Table S1.

Table S1. Mean squared error (MSE) and parameters of the differential freezing spectra n_m (T) obtained using the HUB-backward code and experimental data as input. The reported values were calculated based on the average of three independent runs.

	Number of Populations	MSE	$T_{mode,1}$ (ºC)	s_1 (ºC)	c_1	$T_{mode,2}$ (ºC)	s_2 (ºC)	c2	$T_{mode,3}$ (ºC)	s_3 (ºC)	c3
total	1 (sample 1)	14.4%	-5.9	0.4	1.0						
	2 (sample 1)	3.4%	-10.7	1.2	0.83	-5.4	0.3	0.17			
	3 (sample 1)	0.8%	-9.9	1.0	0.39	-6.3	0.4	0.50	-5.3	0.27	0.11
	3 (sample 2)	0.2%	-10.9	1.1	0.25	-6.8	0.5	0.50	-5.5	0.34	0.25
	3 (sample 3)	1.2%	-12.0	1.0	0.67	-7.1	0.62	0.28	-5.1	0.29	0.05
	3 (FT1 sam. 2)	0.2%	-11.0	1.0	0.23	-6.9	0.50	0.50	-5.6	0.34	0.27
	3 (FT2 sam. 2)	0.2%	-10.4	0.8	0.22	-6.8	0.46	0.50	-5.6	0.35	0.28
	3 (FT3 sam. 2)	0.4%	-10.8	1.1	0.26	-6.8	0.49	0.50	-5.6	0.34	0.24
	3 (FT4 sam. 2)	0.2%	-10.2	1.0	0.27	-6.7	0.48	0.50	-5.6	0.35	0.23
	3 (FT8 sam. 2)	0.2%	-11.8	1.3	0.23	-7.2	0.57	0.54	-5.8	0.40	0.23
	3 (FT12 sam. 2)	0.4%	-9.7	1.0	0.39	-6.9	0.52	0.09	-5.8	0.40	0.52
660 kDa	2	2.0%				-7.0	0.53	0.89	-5.7	0.34	0.11
	3	1.0%	-7.7	0.42	0.63	-6.1	0.41	0.37	-4.7	0.11	0.001
12 kDa	2	2.0%	-8.7	1.20	0.96	-6.0	0.2	0.04			
	3	1.0%	-11.8	0.54	0.35	-8.5	1.2	0.58	-6.4	0.29	0.07

Table S2. Minimum ice nucleating area number of 5.3 kDa protein units that would nucleate ice at a given temperature T_{het}. The minimal areas are computed with the numerical implementation of classical nucleation theory for finite surfaces implemented in the HINT algorithm, assuming that the IN binds ice as strongly as ice itself and that the IN surfaces are squares. The minimum number of protein units is estimated as the ratio of the minimum IN area by 3.8 nm^2, the estimated cross section of a globular 5.3 kDa protein.

T_{het} (ºC)	Minimum Area of Ice-binding Surface (nm^2)	Minimum number of 5.3 kDa units in the functional IN aggregates
-28.15	4	1
-23.15	9	2
-20.55	12.25	3
-18.85	16	4
-15.25	25	7
-13.15	36	9
-12.05	49	13
-10.95	64	17
-9.85	81	21
-8.95	100	26
-8.25	121	32
-7.65	144	38
-7.15	169	44
-6.65	196	52
-6.25	225	59
-5.85	256	67
-5.55	289	76
-5.25	324	85
-5.05	361	95
-4.85	400	105
-4.55	441	116
-4.35	484	127
-4.25	529	139
-4.05	576	152
-3.95	625	164
-3.75	676	178

References

(1) P. Haney, K. Herting, S. Smith, Molecular weight cut-off (MWCO) specifications and rates of buffer exchange with Slide-A-Lyzer Dialysis Devices and Snakeskin Dialysis Tubing, *Protein Biology Application Notes*, Themo Fisher Scientific, (2013)

(2) H. P. Erickson, Size and Shape of Protein Molecules at the Nanometer Level Determined by Sedimentation, Gel Filtration, and Electron Microscopy. *Biological Procedures Online* 11, 32 (2009).

(3) A. Micsonai et al., BeStSel: webserver for secondary structure and fold prediction for protein CD spectroscopy. *Nucleic Acids Research* 50, W90-W98 (2022).

(4) A. Micsonai et al., BeStSel: a web server for accurate protein secondary structure prediction and fold recognition from the circular dichroism spectra. *Nucleic Acids Research* 46, W315-W322 (2018).

(5) C. Cooper, N. Packer, and K. Williams, Amino Acid Analysis Protocols; Humana Press, (2000). DOI: https://doi.org/10.1385/1592590470.
